# What can we learn from the accounts of lay carers administering end of life medications to a loved one at home? Exploring benefits, challenges and ways to empower patients and carers in the future

**DOI:** 10.1017/S1478951525101168

**Published:** 2025-12-22

**Authors:** Annie Hendry, Marlise Poolman, Annmarie Nelson, Stella Wright, Clare Wilkinson, Julia Hiscock

**Affiliations:** 1 North Wales Medical School, Bangor University, Wrexham, UK; 2 Marie Curie Research Centre, School of Medicine, Cardiff University, Cardiff, UK; 3 Betsi Cadwaladr University Health Board, Wrexham, UK

**Keywords:** Lay carers, lay carer administration, home death, unpaid carers, supportive care

## Abstract

**Objectives:**

Most people at the end of life wish to die at home. Lay carers are crucial to supporting a home death and key to a good death is management of symptoms; this may prevent unwanted hospital admissions. If a dying person is too weak to swallow, regular medicines are administered continuously via subcutaneous (SC) cannula. When symptoms “break through,” additional (or as-needed) doses can be given, usually by a visiting healthcare professional. Delayed symptom control can occur due to time taken for healthcare professionals to arrive at the home.

Lay carers can be trained to administer as-needed SC medicines; the practice is safe and legal in the UK, although not widely used. The “CARer-Administration of as-needed SC medication for breakthrough symptoms in people dying at home” (CARiAD) feasibility trial of lay carer administration in the UK was the first to conduct in-depth interviews with carers trained in the practice.

The objective of this paper is to give voice to carers and show how experiences reflect benefits and challenges of lay administration at the end of life.

**Methods:**

Qualitative interviews with carers trained in the practice. Interviews were analyzed using Interpretive Phenomenological Analysis.

**Results:**

Caring for a loved one at home during the last days of life is complex. Accounts reveal a desire to fulfill a loved one’s wishes by keeping them at home and having the death they wanted. Carers were afraid of uncontrolled symptoms, especially pain, empowered by the ability to help and grateful to avoid long delays. Potential for carer burden and fears of hastening death require careful reassurance from health care professionals.

**Significance of results:**

We learned that carers endorsed and embraced the opportunity to do more to keep their loved ones comfortable and at home. This is significant in making the case for wider access to the practice in the UK.

## Introduction

Most people entering end of life care express a wish to die at home, although only around half of those achieve it (Poolman et al. 2019). The home setting usually refers to the primary place of residency and can be seen as a place of comfort and safety and staying at home during the dying phase can allow the patient and their family to preserve a sense of the “normal life” (Horsfall et al. 2017). The support of a home death is a significant task for health care professionals (HCPs) who work to care for patients at home and avoid unwanted hospital admission (Hoare et al. 2019). Lay caregivers (i.e., family or friends of a dying person providing informal/unpaid care) are crucial to facilitating a home death and, without the support of a lay carer, many patients are unable to die at home (Rowland et al. 2017; Hoare et al. 2019; Becqué et al. 2021a). A key predictor of a person having a home death is the presence of a lay carer (Johnson et al. 2021). Good communication between HCPs, patients and families about the end of life can facilitate decision making, however, uncertainty regarding prognosis can mean that preferences for care are not discussed and therefore not always met (Sleeman 2013).

A key component of supporting a home death is to control and manage symptoms as effectively as possible (Johnson et al. 2021). Common symptoms which may occur in the last days or hours of life are pain, nausea/vomiting, noisy breathing, restlessness/agitation and breathlessness (Teunissen et al. 2007). These symptoms are continuously managed via subcutaneous (SC) infusion using a syringe driver. Additional (or as-needed) doses may be needed for symptoms that “break through” (Poolman et al. 2019). Lay caregivers take on many tasks, including assisting with oral medication for breakthrough symptoms, however, as the dying person loses the ability to swallow, SC medications will need to be administered. Usual practice for as-needed SC medication is administration by an HCP such as a District Nurse (DN) (Poolman et al. 2019).

Medication for breakthrough symptoms is prescribed in advance and then stored in the patient’s home to be used by the visiting HCP (Poolman et al. 2019). Delays can occur due to HCPs availability and travel time to patients’ homes, especially when the visits are out of hours or the home is in a rural location. Long waits can mean that symptoms are not adequately managed and result in distress for patients and carers (Poolman et al. 2019). The training of lay carers to administer SC medication for common breakthrough symptoms can offer a solution to these delays, improve symptom control and minimize distress. Lay carers are often ideally situated to give these medications as they are in close proximity and can become “experts” in the care of their loved one as the people who know them best, particularly as the patient loses ability to communicate verbally (Crowther et al. 2022). The use of SC medication by lay carers is safe and legal in the UK although it is not common practice everywhere and many patients and carers are unaware that it may be an option (Poolman et al. 2025).

Lay caregivers may welcome the opportunity to be more involved in supporting their loved one at the very end of life due to a desire to fulfill the wishes of the dying person to remain at home (Lobb et al. 2019). Carers can find a sense of empowerment in having more control over their situation and spending more time with their loved one at the end of life (Lobb et al. 2019; Spelten et al. 2019). Other studies have reported caregivers’ desire for their loved one to “die with dignity” and that being able to facilitate this had a positive impact on their own bereavement (O’hara et al. 2023). It has also been shown that achieving the preferred place of death of a loved one is of significant importance to lay carers (Pollock et al. 2024).

Whilst many people find being a caregiver rewarding, there are substantial challenges involved. Lay caring can require a huge investment of time and resource and be physically and emotionally demanding (Hudson et al. 2018; Hoare et al. 2019; Becqué et al. 2021a). Some carers may be naïve about what the dying process can involve and the realities of caring for a dying person (Goldblatt et al. 2019; Hoare et al. 2019; Spelten et al. 2019). Carer burden can increase as the patient becomes increasingly dependent and relationships between family members can also suffer under the pressure (Goldblatt et al. 2019). Lay caregivers may struggle with symptom control and fear administering pain medications due to misinterpretation of symptoms, fear of overdose, side effects or hastening death (Lobb et al. 2019; Johnson et al. 2021). HCPs can play an important role in supporting the lay carer with both practical and emotional needs (Johnson et al. 2021; Becqué et al. 2021a).

The “CARer-Administration of as-needed SC medication for breakthrough symptoms in people dying at home” (CARiAD) study was a randomized controlled feasibility trial to assess acceptability and feasibility of lay carers administering SC medications to loved ones dying at home (Poolman et al. 2020, 2025). Carer voices are missing from current research and, as part of this study, in-depth qualitative interviews were conducted to capture carer perspectives and carers were given the opportunity to reflect on their experiences and tell their stories in their own words. Carer accounts are essential in understanding the practice in real world settings and can inform future policy and facilitate improved access to the practice for those who can benefit. Little is known about carer experiences of lay administration in the UK and these nterviews were the first to explore, in depth, the experiences of the practice in the UK setting.

The paper is cognizant of the fact that the practice has been implemented in some areas in the UK since the completion of the CARiAD study at the end of 2019. As such, it reports in detail the findings from the qualitative interviews in relation to carer experiences of the practice in the UK setting. It focusses on the benefits lay carers perceived, the challenges they faced and where future research in avoiding potential carer burden and communication regarding the practice might aim to make lay carer administration more widely available to meet the needs of patient and carers.

## Methods

### Interviews

Semi-structured qualitative interviews were conducted with 10 carers from the intervention arm of the CARiAD trial post bereavement. All were trained in the practice at home by either a district or specialist palliative care nurse, although not all of them gave injections once trained. Interviews lasted approximately 60 minutes. All participants were provided with a Patient Information Sheet and written informed consent was taken at the time of interview. Interviews were carried out in participants’ homes, at the research center if requested by the carer, or by telephone. All interviews were audio recorded and fully transcribed.

### Purposive sampling

Patients and carers were recruited to the study between January 2018 and March 2019 (Poolman et al. 2019). A sample of lay carers were contacted by telephone two to four months post bereavement. Sampling criteria used were geographical location, gender, and rurality. All carer names in this paper are pseudonyms to ensure participant confidentiality.

### Analysis

Interview data were analyzed by the same researcher who carried out the interviews using Interpretive Phenomenological Analysis (IPA) (Smith and Fieldsend 2021). The interviews were designed to explore in depth the views of carers and the meaning that administering the SC medication had for them. IPA was chosen as the most appropriate method of analysis as the interviews sought to explore personal accounts, views and experiences (Smith and Fieldsend 2021). The analysis followed the stages of IPA beginning with immersion in the data, initial noting of the transcript and the development of themes (Smith et al. 2009). Themes were grouped according to connections between them, this was repeated across all transcripts and patterns across cases were identified to form a thematic structure (Smith et al. 2009). Rigor in IPA is ensured by iterative and close reading of transcripts and listening to recordings, discussing interpretations with the research team and keeping a reflexive journal keeping note of decision making throughout the process (Vicary et al. 2017; Nizza et al. 2021).

## Results

The results from the interviews are presented thematically below.

### Learning about lay carer administration

#### On introduction to the practice

Carers were asked to discuss their first thoughts when HCPs offered them training for lay carer administration. Some reported initial hesitancy, which diminished with further explanation of how the administration would work. Several carers described feelings of surprise as they had never considered the possibility that carers could administer these kinds of medications. There was also relief at the realization that there was something more they could do to help keep loved ones at home.

Timing for introducing lay carer administration is complicated due to difficulties of prognostication for the last days of life. Carers of patients who deteriorated at a slower rate had more time to prepare for the prospect of administering the medications and were more likely to describe their approach as at the right time. Carers of patients who experienced a more rapid deterioration reported that they would have preferred to have been approached earlier than they were. Reasons for this included, as described by one carer, wanting to have more time to prepare.

I think it might’ve been, might’ve been better if it’d come a little bit earlier because [Husband] had already gone downhill quite a bit and it was virtually er, you know, the syringe drivers were already – they hadn’t been put up yet but the plan was soon and so he’d already deteriorated and…. I think if it had been a little bit earlier it might’ve been better.

Hillary, urban

#### Training for lay carers

The training given to carers was largely described as having been clear and helpful, however, different carers required different amounts of training to feel confident. Some carers seemed to feel confident after only one training session, particularly those who were comfortable around medications already such as David who had been caring for his wife and managing medications for many years. Other carers such as Ffion and Josephine, who were both HCPs themselves also found the training straightforward and easy to understand. However, other carers reported that they required more than one training session. One carer had had several sessions but would have liked more to feel confident, however, she also explained that she never needed to administer the medication and seemed relieved that was the case.

I’d only had it explained to me about a couple of times. Now, they would have done more, but he died. So, there was no point in carrying on that side of it, But I think I probably had enough confidence to have someone on the phone to actually do it. And if not, you just pray and you do it. It’s amazing what you can do when you have to.

Myra, rural

Carers described their experiences of using the equipment to administer the medications. Whilst carers expressed initial apprehension about the use of medical equipment, they reported feeling more confident following training and using the practice kits provided. Two carers reported issues with dexterity, one described how he had difficulty opening ampoules early on but had been reassured by a DN that many people experienced difficulty with ampoules. The second found the equipment to be quite technical and would have liked more training had she gone on to administer any medications.

It was the initial ampoule, breaking one of those ampules, big clumsy hands, but after that when I made the first mistake, I was fine. You realise, because I’d never used an ampoule before. Broke that, “Oh god!” [Laughs] Throw that one away. The team said it was quite a common thing when you’re not used to that.

David, urban

#### Understanding needle-free administration

The participants’ accounts show that carers have concerns about giving injections as the word “injections” conjures up images of needles, which, in turn, raises fears over making mistakes and potentially causing harm. Several carers reported feelings of relief when they understood that they would not have to use needles or break the skin. This was often linked to the fact that they wanted to relieve pain, not potentially cause pain. One carer described her husband as having a fear of needles and explained that part of their desire to take part was so that she could give medications rather than him have more doses from an HCP, when they both realized that the administration involved no-needle injections they were greatly comforted. One carer, who was a retired doctor, specifically stated that she was glad she did not have to “stick needles” into her husband.

The difference for me was the emotional side, I think, because with a patient, you’re desperately wanting to make the patient feel better. I also wanted to make my husband feel better. Um but sticking a needle into your husband is a little bit different from-from a patient because of the emotional involvement.

Josephine, urban

### Fears and concerns

#### Symptom recognition and management

The ability to recognize breakthrough symptoms were of concern to some carers. As management of symptoms depends on recognizing them, carers worried about leaving loved ones in pain without realizing. Recognizing the difference between symptoms, such as pain and agitation, was sometimes difficult, particularly if the patient was no longer able to communicate verbally. Some carers described consulting with other relatives about whether to give medications. Carers gained confidence in recognizing breakthrough symptoms once they had administered their first dose or had been reassured by either family members or HCPs.

I was glad that my son was here as well. He-he’s not medically trained at all but… I asked him, “Oh, do you think Dad should have something now?” And I valued his input, really, because he said, “Yes, definitely.” You know, “Yes, yes, yes. He’s getting a bit restless now.” Or, “His breathing is not so good, you know, and if we can relieve that.

Josephine, urban

All of the carers wanted to help their loved one avoid uncontrolled symptoms, particularly pain and this was a key factor in deciding when to administer.

That you weren’t scared of the uncontrollable pain or severe sickness or something that might’ve been there. That-that gave us comfort – comfort – and confidence, I would say, to keep my wife at home, rather than, you know feeling that you had to take her to a hospice to get her symptoms relieved.

George, rural

One carer reported being upset during times when symptoms were uncontrolled as he felt that HCPs were blaming him for being unable to manage the symptoms with oral medication. The desire to be able to manage and control symptoms as they occurred was a major contributor to wanting to administer.

So, yeah, it was just pain relief, you know, in my mind if anything I was doing was gonna relieve it then it was worth doing so I didn’t think of anything else than it was gonna alleviate any pain or suffering for my mum.

Tom, urban

#### Long waiting times

There was also fear of long waiting times for HCPs to arrive at the home to relieve symptoms. Long periods of waiting for help to arrive, were a source of concern for all carers, especially those who lived in rural locations. There was a common feeling throughout the accounts that waiting times seem even longer at night and these long waits during sleepless nights contributed to feelings of powerlessness and isolation.

As I said before, it’s just positive. I was not reliant on waiting for the team to come out to give injections to relieve any symptoms she might have with the vomiting and the anxiety and the pain relief. So that was all positive, not having to wait. It was -. I could give it as soon as it was required.

David, urban

#### Causing harm or hastening death

Concerns about making mistakes with medication were raised throughout. All carers expressed some concerns about the potential for error. A big concern for carers was the possibility of causing or hastening death. While carers knew that their loved ones were in the last stages of life, there was a desire not to contribute to that in any way and there was a fear among carers of what could happen were they to give an injection and the patient died very soon after – they did not want to take what little life their loved ones had left away. There was also a concern that they could be held accountable or be blamed if the patient passed away after medicines that they had administered.

I was concerned if I was to administer morphine and he, say, passed away 10 minutes later, could I be held accountable … that I’d done something slightly wrong?

Claire, rural

### The benefits of lay carer administration

#### Wish fulfillment and a “good death”

The desire to have a home death was something that carers all reported to be the wishes of their dying loved one. The importance of a home death was presented in a variety of ways by carers, as a more comfortable environment, a place in which family and pets could be present at all times, and one over which the patient was able to have more control.

Thought of her being in her own bed, surrounded by her own pictures and books and music and, you know, the views. We live in a lovely place and the views and the noises and familiarity of home gave us comfort, really.

George, rural

Lay carer administration was seen as an extra way of ensuring that a home death could occur. This was a driving force for many carers who were seeking above all to fulfill the wishes of their loved one.

And they were all saying that (husband’s) wish was the most important … to remain in his home, they could see it was so important. And it’s that reason, you know, obviously I did it … the hardest thing I’ve ever done.

Claire, rural

It may be that some lay carers put the wishes of their loved one to stay at home above their own concerns regarding administering medication and being the main carer. One carer whose husband was admitted to the hospice stated that she felt a great sense of guilt and regret at not having been able to keep him at home despite the circumstances of his admission being out of her control.

Some of the carers had previous experience of caring for, or being with, people who were in the last days of life. Previous experiences seemed to have influenced the desire to administer especially if during those previous times they had experienced long delays or uncontrolled symptoms. Carers throughout the interviews explained that they did not want their loved one to be in pain or to suffer as they had seen happen to others in the past. Lay carer administration seemed to be a way of helping and trying to ensure that a loved one was able to have a “good death.”

Well, I had seen people die before and I wanted him to have the best kind of death. I really did.

Josephine, urban

#### Carer and patient empowerment

Carers found a sense of purpose in being able to manage and administer medications rather than taking a more traditional passive role. This led to increased feelings of empowerment and control. Some carers reported a sense of personal peace in knowing that they were able to do all they could for their loved one during the last days of life. One carer described how he missed his wife greatly but was content in the knowledge that he had done his best for her. Lay administration was reported to have been empowering for patients, giving them more control over their own death, rather than having decisions made for them.

So, those were two really important things for her and CARiAD was part of that, I think. “These are things, I can make decisions about. I can’t not have cancer. I can’t not die. But I can make decisions about how that happens”. And that, I think, was really important to her.

Laura, urban

## Discussion

These findings give a valuable insight in to the experiences of people caring for a loved one at home in the last days of life and are the first of their kind in the UK context. The interviews showed the complexities of lay carer experiences, the things salient to them, and the ways in which carer administration can support a home death. Whilst there are many factors contributing to a good death, it may be argued that dying at home as a place of choice could be key to facilitating wishes such as being near family and being free from anxiety (Delgado-Guay et al. 2016). Carers have been shown to need timely access to information, so they have time to understand and prepare for what is involved (Flemming et al. 2019). The carers in this study reported feelings of surprise that lay carer administration was an option, some felt that having more information earlier would be beneficial, however, as reported in previous work, difficulties with prognostication can affect timing of discussions (Sleeman 2013).

The amount of training needed varied between carers but the training and practice kits increased confidence and the realization that administration was needle-free eased anxiety around injections. The findings correlate with other studies which show that training must be flexible and adaptable depending on the needs of the lay carer and that some carers will need to be trained more than once (Poolman et al. 2020; O’hara et al. 2023). The needs of carers may also vary according to family structure and living situation, and this may be facilitated in the future by training multiple carers to administer where possible (Becqué et al. 2021b). Carers will need emotional support from HCPs as well as practical advice and training as caring for a dying loved one can be an emotional time and the addition of managing medications may add to carer duties and emotional burden (Becqué et al. 2021b).

Symptom management, especially pain control, was often cited as the main reason carers in this study wished to take part. It has been shown that family members may struggle to cope when symptoms are uncontrolled and that there is a need for better communication from HCPs about how symptoms may present (Spelten et al. 2019; Bowers et al. 2024). Other studies have reported carer distress when observing certain symptoms, especially pain or severe agitation, carers may also be frightened by noisy breathing, particularly when unprepared for the sound (Hendry et al. 2022). HCPs should be aware that carers are inexperienced and that seeing and hearing the dying process can be a major burden (Lobb et al. 2019). Carers who manage medications can be fearful of causing pain or hastening death (Lobb et al. 2019). The carers in this study expressed similar concerns regarding misinterpretation of symptoms and last injections, however, they reported ways in which they overcame this, including seeking affirmation of symptoms with other family members or asking advice from HCPs (Johnson et al. 2021). It may be that carers in this study became “experts” in caring for their loved ones and, as they knew them so well, were able to interpret symptoms through nonverbal communication and therefore needed HCP support less often over time (Crowther et al. 2022).

Carers in rural or remote areas have expressed feelings of isolation and lack of support during the caring process (Spelten et al. 2019). In this study, carers were concerned about delays in controlling symptoms and the potential for long waits for HCPs to arrive, particular for those who lived in rural locations. The ability to administer medication themselves was empowering and gave a sense of control, alleviating the fear of delay.

Above all, carers express a desire to fulfill the wishes of the patient by keeping them at home and to facilitate a “good death” (Lobb et al. 2019; Spelten et al. 2019; O’hara et al. 2023). Carer journeys in the CARiAD study were not always straightforward and carers reported times of distress and uncertainty, however, many also expressed a sense of peace in having fulfilled the wishes of their loved ones and in the knowing that they had done all they could.

## Conclusion

Accounts of carers revealed the complexity of issues surrounding caring for a loved one at home during the last days of life. Accounts also reflect carers’ experiences, both practical and emotional, of administering SC medications. The desire to fulfill the wishes of loved ones who want to have a home death was paramount to carers. However, it is possible that patient wishes may overshadow carer needs and it is important to ensure carers are well supported and not coerced by patients or overburdened by responsibility. The acceptability of administering SC medication was driven by the desire to control symptoms as they occurred, particularly in rural areas where delays may be longer. Carers reported feeling empowered by the ability to assist with symptom control rather than experience the feelings of powerlessness that may come with waiting for HCPs. A key concern for carers was the potential for causing harm or hastening death, highlighting the importance of robust training in the practice and ongoing reassurance from HCPs. Overall, the findings from this study indicate that carers in the UK endorse and embrace the opportunity for this extension of the caring role and that in future this may help to de-medicalize the dying process. These findings amplify those of the feasibility trial, which suggests this practice is widely endorsed by lay carers (Poolman et al. 2020, 2025).

### Study strengths and limitations

This was an original study that employed a rigorous methodology to gain insight into carer experiences. These are the first interviews with carers trained in this practice in the UK. This practice has potential to benefit a much wider population, and the knowledge of carer experiences and preferences will serve to improve access and options for home-based palliative care. This practice has now been implemented in Wales.

A limitation of the study was that some carers were no longer contactable post bereavement and could not be invited to interview. The carers in this study were trained as part of the wider trial and further research may be needed to ensure findings are applicable to other areas of the UK.
